# Southend and Its Medical Officer

**Published:** 1907-09-14

**Authors:** 


### The Hospital

A JOURNAL OF

**The Medical Sciences and Hospital Administration.**

NEW SERIES. No. 25, VOL. I. [No. 1097, VOL. XLII.] SATURDAY, SEPTEMBER 14, 1907.

### SOUTHEND AND ITS MEDICAL OFFICER.

THE position adopted by the Municipal Council of Southend in relation to the medical officer of health of the borough, emphasises very emphatically the need for the modification of the power of control which local health authorities at present exercise over their medical officers. It appears that by the conditions of Dr. Nash's appointment, his second triennial term of service came to an end in August last. At the meeting of the Town Council in that month a motion was made for his reappointment for a further term of three years at the same salary as before—namely, £600 per annum. To this an amendment was moved reducing the salary to £500 and limiting the appointment to one year only. This amendment was carried, and was subsequently adopted as a substantive motion. For the change thus made no member of the Council ventured in any way to question Dr. Nash's ability or to reflect upon the efficiency of his work. Indeed, it is beyond dispute that the public health and sanitary position of Southend have in a very high degree benefited by Dr. Nash's services. Testimony from the local medical profession and from other influential inhabitants leaves no room for doubt on this point. Hence the conclusion is inevitable that personal reasons, the nature of which it is not difficult to imagine, prompted the attempt to disparage his work and to reduce his salary. Those concerned in this attempt, however, seem to have been very ignorant of their powers. It was beyond their authority to alter the terms of the appointment without having first communicated with the Local Government Board; nor could an election be conducted under the new conditions until the vacancy had been announced by advertisement. Hence, even had Dr. Nash been willing to accept the new terms—as a matter of fact he refused them—his election would have been invalid. Thus, as a result of the action of its Town Council, Southend found itself without any medical officer of health, and the Council had to beg Dr. Nash to act for three months while permanent arrangements could be made. These proceedings have very properly brought from the Local Government Board a demand for an explanation, the nature of which will be anticipated with interest. For the present it is sufficient to note the position in which an efficient and zealous officer and the care of the public health may be placed by local worthies endowed with municipal control, but ignorant both of their powers and of their responsibilities.

Of the bungling which characterised the proceedings of the Council little need be said. It concerns mainly the constituents by whose votes the members of the Council are placed in their present position. These, however, may be asked to note the manner in which the Council proposed to treat an officer whose efficiency and zeal in the public interest no one dared to question. To have voted against Dr. Nash's reappointment would at least have been a straightforward course, even though no adequate reason would have been advanced for such a proceeding. But to endeavour indirectly to depreciate his position as a public servant can only be characterised as an unworthy and contemptible action. And the constituents of the worthies responsible for this line of policy may estimate the efficiency of their representatives by observing that the action decided on was outside the law, left the borough without a medical officer of health, and has secured for the Town Council the attention of the Local Government Board.

The action of the Southend Council raises still wider issues. Medical officers of health have large responsibilities, and in the discharge of these neither fear nor favour must be exhibited. It has repeatedly been shown that such public duties may conflict with the private interests of members of the local authority. Hence it becomes a question whether the position of the medical officer of health can be safely and wisely left in the control of the local authority. The claim of justice to the individual is by no means to be neglected. But setting that aside for the time being, the larger interest of the entire local community must be borne in mind. To protect this the medical officer of health must be undeterred by the frowns of those in authority. He must, in short, enjoy security of tenure, qualified only by appeal to the Local Government Board. The action of the Southend Council will help to the attainment of this end, and in the meantime the Council may be confidently left in the firm grasp of the central authorities.

